# Eccrine porocarcinoma of the lower extremity: A case report and review of literature

**DOI:** 10.1186/1477-7819-9-94

**Published:** 2011-08-22

**Authors:** Oliver Chang, Ashraf Elnawawi, Bernard Rimpel, Armand Asarian, Nadeem Chaudhry

**Affiliations:** 1 General Surgery Department, The Brooklyn Hospital, Brooklyn NY, USA; 2 Chief of the Pathology Department, The Brooklyn Hospital, Brooklyn, NY, USA; 3 Gernal Surgery Department, The Brooklyn Hospital, Brooklyn, NY, USA; 4 Chief of the Colorectal Department, The Brooklyn Hospital, Brooklyn, NY, USA; 5 Chief of the Plastic and Reconstructive Surgery Department, The Brooklyn Hospital, Brooklyn, NY, USA

**Keywords:** eccrine porocarcinoma, sweat gland tumor, malignant eccrine poroma

## Abstract

Eccrine porocarcinoma is a rare malignancy of the eccrine sweat gland. It is usually found frequently on the lower extremities, and it affects both sexes equally usually in the sixth to seventh decade. In our case, we present a 42-year-old male patient with a recurring exophytic tumor on the right lower extremity without local extension. The initial tumor was biopsied, excised and diagnosed as an eccrine poroma. The tumor then recurred 6 years later, was re-excised, reconstructed with a soleus muscle flap and diagnosed as an eccrine porocarcinoma.

## Background

Eccrine porocarcinoma (EPC), a rare malignant sweat gland tumor, representing only 0.005% of epithelial cutaneous neoplasms. The first reported case, in 1963, was attributed to Pinkus and Mehregan [1], coined as "epidermotropic eccrine carcinoma", and since then, presentation have been limited due to the rarity of this tumor. It was only a few years later, the term "eccrine porocarcinoma" was introduced by Mishma and Morioka in 1969 [2]. These lesions are most commonly found on the lower extremities, followed by the head, scalp, upper extremities, trunk and abdomen [1,3-11]. The tumor can arise from the intraepithelial portion of the eccrine sweat gland, the acrosyringium, being a primary tumor or, even more common, a malignant transformation of an eccrine poroma (EP). In this case, we present a 42-year-old patient with a right lower extremity mass that was previously diagnosed with a benign poroma later, over 6 years, found to have had malignant transformation.

## Case Report

A 42-year-old male presented with an exophytic mass on the right lower extremity that had developed over the last 6 years. The patient denied any type of trauma to the area or insect bites. The mass had been previously excised and pathology showed a benign poroma. The laboratory work-up for this patient was within normal limits.

**Figure 1:**
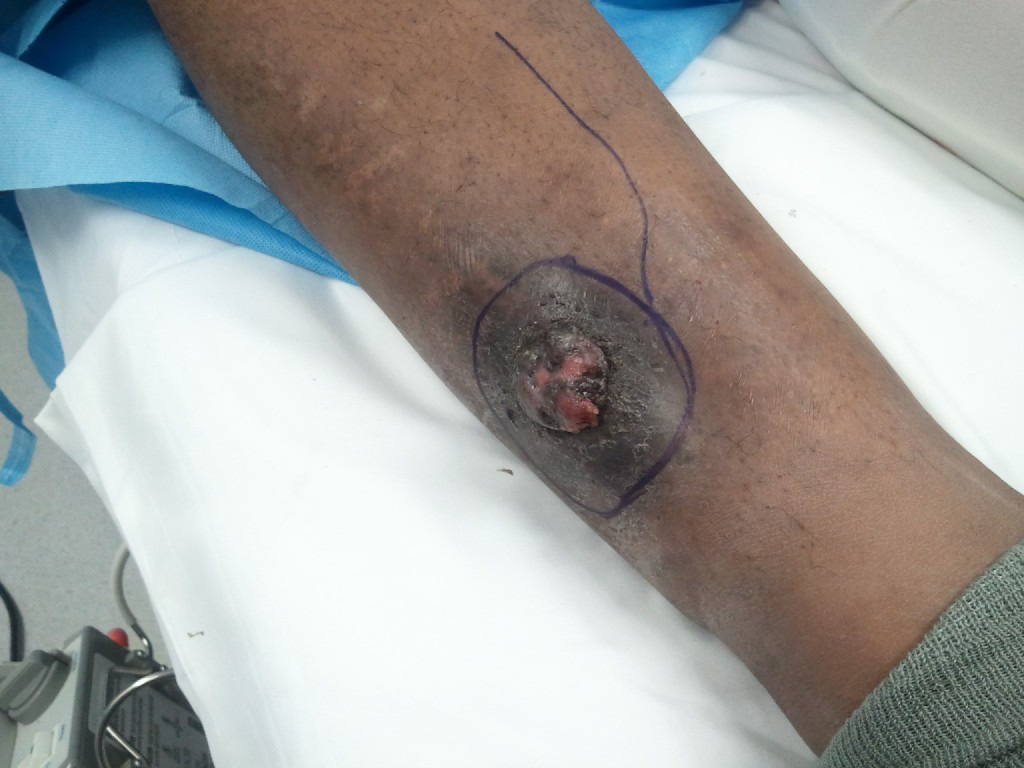
**Preoperative image of the pink exophytic outgrowth - 2.0 × 3.0 cm**.

On physical exam, there were no lymphadenopathy in the inguinal, axillary or clavicular areas were noted. The tumor itself measured 3.0 × 2.0 cm with a width of 0.6 cm (Figure 1). Pink exophytic, fungating outgrowths are a signature to this tumor. In combination with the patient's previous history of benign poroma, the treatment of choice was a wide local excision of the mass with 2.5 cm margins - both circumferentially and deep to the level of the periosteum. A soleus muscle flap was then raised and used to reconstruct the defect. The muscle was then covered with a split thickness skin graft.

**Figure 2:**
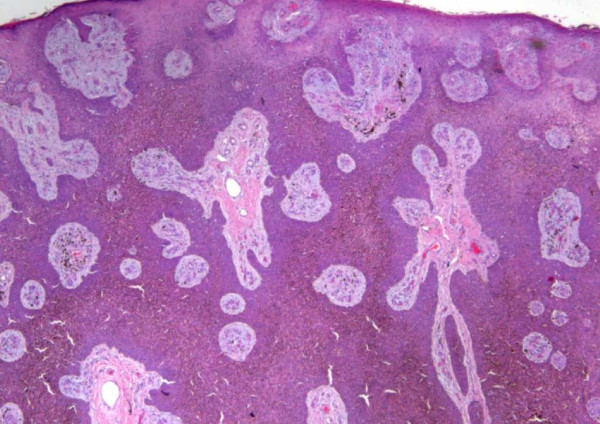
**The tumor cells are in-situ and invasive**. Malignant eosinophilic lobular masses or islands are seen with eosinophilic cells that are polyhedral and fusiform that have variable cytoplasm, hyperchromatic nuclei and indistinct cell boundaries.

**Figure 3:**
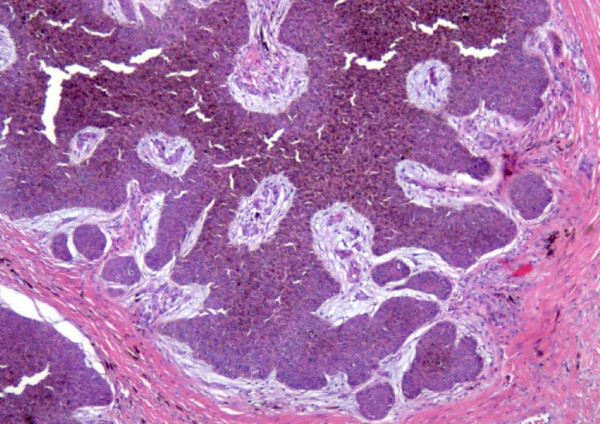
**High power view show invasive carcinoma cells**. There is obvious cell atypia with many mitotic figures seen. The squamous cells have variable differentiation accompanied with changes in pigmentation that are aligned in a horizontal nodular pattern. Both carcinoma in situ and invasive component noted.

The macroscopic examination of the specimen showed an exophytic mass measuring 3 cm in diameter. Microscopically, the specimen displayed malignant eosinophilic lobular masses with eosinophilic polyhedral fusiform cells that contained variable cytoplasm, hyperchromatic nuclei, distinct nucleoli and indistinct cell boundaries (Figure 2). There was obvious atypia and frequent mitotic figures, epidermotropism, variable squamous differentiation and cell changes with pigmentation in a horizontal nodular pattern. Both carcinoma in situ and invasive components were noted (Figure 3).

**Figure 4:**
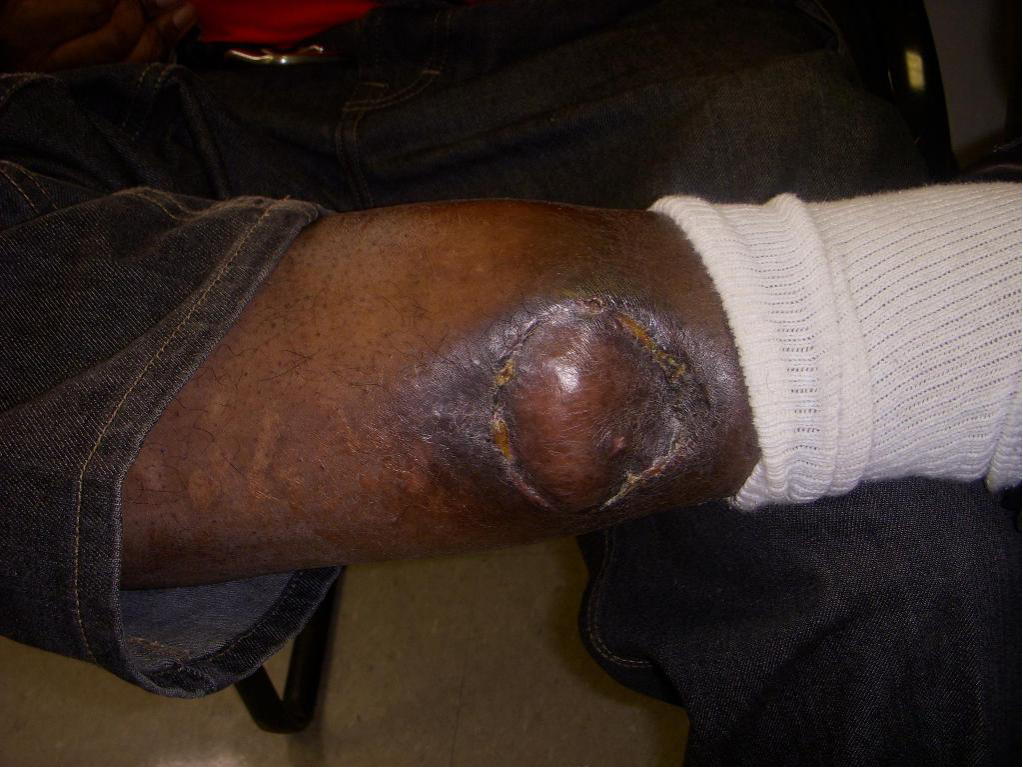
**Postoperative image of the excised area with a soleus muscle flap reconstruction**.

The final pathology report stated the specimen was a porocarcinoma in situ with clear surgical margins. During the 6 month and 1 year post-op follow up, the wound had healed without any complications or recurrence (Figure 4). The physical exam remains unchanged with no palpable regional lymph nodes.

## Discussion

Eccrine porocarcinoma (EPC) is more common in elderly patients and most cases occur in the sixth to seventh decade of life. In our case, we presented a male in his early 40 s and with time, has become malignant from a previous benign poroma. Men and women are usually equally affected, and prevalence varies in both sex in diverse studies [12-14]. Approximately 50% of EPC appear first in the lower limbs; in that, more than 40% occur below the knees [15]. Less than 5% involve the scalp [16]. Other affected sites are face, ear, eyelids, abdomen, vulva, penis and pubis.

Theorectically, eccrine porocarcinoma progresses from benign to malignant. This is supported clinically by long histories, mean 8.5 years and recent onset of rapid growth in longstanding cases. Only a period of 6 years duration was noted in our case. Histological reports of dysplasia and malignant change in benign variants, including eccrine poroma and hidroacanthoma simplex support this theory [17].

The pathogenesis and roles of possible pre-invasive precursors of this lesion is still unknown [18]. It seems that the upper portion of the dermal eccrine duct could have a role in the oncogenesis. More recently, the p53 gene involved in tumor suppressing, could be involved in EPC carcinogenesis [19]. Throughout the literature search, there is not a definite time line in which a poroma advances and becomes a porocarcinoma.

Treatment of choice for EPC is total surgical excision with broad tumor margins and regional lymph node disection if involved. There is still insufficient literature data on cryosurgery and electrosurgery in order to accurately assess indication and tumor recurrence [18]. Chemotherapy with methotrexate, cisplatin, adriamycin and bleomycin or isotretinoin and interferon alpha have been used with partial or little response. Radiation therapy and chemotherapy seem to be ineffective to control tumor recurrence or metastasis [16]. In our case, post-op chemotherapy or radiation was not given to the patient. There is no effective definitive treatment available for EPC at the moment but surgical excision is still the treatment choice.

It is important to have this diagnosis as a differential for cutaneous lesions due to the malignant nature of the tumor if left unattended. EPC must be considered in the differential diagnosis of patients older than fifty years with long standing tumors in the limbs and head - including basal cell carcinoma, Paget's disease, melanoma and metastatic cancer. EPC has been documented to occur even in the nail fold of the toe mimicking an ingrown toe nail. Biopsy is indicated and, even with a previous diagnosis of EP, one must be attentive to risk of malignancy in the future or if it is a recurrence [20]. EPC is a rare malignancy of the eccrine sweat glands but yet curable if accurately diagnosed and properly treated. Close follow-up to detect local recurrence and lymph node metastasis is recommended since further surgical intervention may be curative.

## Conclusion

Eccrine porocarcinoma (EPC), a rare malignant sweat gland tumor, representing only 0.005% of epithelial cutaneous neoplasms. Being only 0.005% of all cutaneous neoplasms makes this diagnosis intricate, but not impractical if more literature is provided of this rare pathology. The treatment is a multidisciplinary effort that consists of a pathologist and surgeon. And if treated in the early stage, surgery can be curative. EPC is a rare malignancy of the eccrine sweat glands but yet curable if accurately diagnosed and properly treated. Due to the limited amount of literature and cases documented, it is important to have a basic knowledge of this disease process as a differential diagnosis when dealing with cutaneous lesions.

## Consent

Written informed consent was obtained from the patient for publication of this case report and accompanying images. A copy of the written consent is available for review by the Editor-in-Chief of this journal.

## Competing interests

The authors declare that they have no competing interests.

Non-financial competing interest: The authors declare that they have no competing interests.

## Authors' contributions

OC wrote the article and participated in the collection of data in the literature review. NC performed the surgical procedure and acted as preceptor for the project. AE performed the microscopic analysis of the surgical specimen and provided the pathological slides with description. BR performed originally surgery and acted as preceptor and gave final approval for publication. AA acted as preceptor and gave final approval for publication. All of the authors have read and approved the final manuscript.

## References

[B1] Pinkus H Mehregan AH Epidermotropic eccrine carcinoma Arch Dermatol 1963 88 597 606 PubMed 14060075 10.1001/archderm.1963.01590230105015

[B2] Mishma Y Morioka S Oncogenic differentiation of the intra-epidermal eccrine sweat duct: eccrine poroma, poro-epithelioma, and porocarcinoma Dermatologica 1969 138 238 50 10.1159/000253989 5771637

[B3] Orella JAL Penabla AV Juan CCS Nidal RV Morrondo JC Alvarez TT Eccrine porocarcinoma: report of nine cases Dermatol Surg 1997 23 952 8 9357503

[B4] Akioshi E Jogita T Yamaguchi R Toyoda H Kawashima M Hidano A Eccrine porocarcinoma Dermatologica 1991 182 239 42 10.1159/000247804 1653154

[B5] Goedde TA Bumpers H Fiscella J Rao U Karakousis CP Eccrine porocarcinoma J Surg Oncol 1994 55 261 4 10.1002/jso.2930550413 8159010

[B6] Glimme H Petres A Brgen E Wiemers S Schopf E Vanscheidt W Metastasizing porocarcinoma of the head with lethal outcome Dermatology 1999 198 298 300 10.1159/000018135 10393458

[B7] Grishkumar H Kamineni S Hwang RR Levy J Sadler R Eccrine porocarcinoma Dermatol Surg 1997 23 283 4 10.1111/j.1524-4725.1997.tb00692.x 9236878

[B8] Weedon D Strutton G Tumours of cutaneous appendages: skin pathology 1998 London: Churhill-Livingstone 746

[B9] Kolde G macher E Grundmann E Metastasizing eccrine porocarcinoma: report of two cases with fatal outcome Pathol Res Pract 1991 187 477 81 1652130 10.1016/S0344-0338(11)80010-5

[B10] Snow S Eccrine porocarcinoma, commentary Dermatol Surg 1997 23 584

[B11] Robson A Greene J Ansari N Kim B Seed PT McKee PH Calonge E Eccrine porocarcinoma (malignant eccrine poroma): a clinicopathologic study of 69 cases Am J Surg Pathol 2001 25 710 20 10.1097/00000478-200106000-00002 11395548

[B12] Poiares Baptista A Tellechea O Reis JP Cunha MF Figueiredo P Porocarcinome Eccrine - Revue de 24 cas Ann Dermatol Venereol 1993 120 107 15 8338322

[B13] Ruffieux C Ramelet AA Porocarcinoma eccrine Dermatologica 1985 170 202 6 10.1159/000249532 2987051

[B14] Walsh MS A case of eccrine poroma J R Soc Med 1990 83 529 30 2172536 10.1177/014107689008300819 PMC1292784

[B15] Berke A Grant-Kels JM Eccrine sweat gland disorders: part I - neoplasms Int J Dermatol 1994 33 79 85 10.1111/j.1365-4362.1994.tb01532.x 8157406

[B16] Okada N Ota J Sato K Kitano Y Metastasizing eccrine sweat gland carcinoma Arch Dermatol 1984 120 768 9 10.1001/archderm.120.6.768 6721544

[B17] Pylyser K De Wolf-Peeters C marine K The histology of eccrine poromas. A study of fourteen cases Dermatologica 1983 167 243 9 10.1159/000249790 6317476

[B18] Turner JJ Maxwell L Bursle GA Eccrine porocarcinoma: a case report with light microscopy and ultraestructure Pathology 1982 14 469 75 10.3109/00313028209092129 6296749

[B19] Akalin T Sen S Yuceturk A Kandiloglu G P53 protein expression in eccrine poroma and porocarcinoma Am J Dermatopathol 2001 23 402 6 10.1097/00000372-200110000-00003 11801771

[B20] Fabiane A Maira M Carlos A Ezio A Jesus R José F Eccrine porocarcinoma: report of four cases and literature review An Bras Dermatol 84 no.5 Rio de Janeiro Sept./Oct. 2009

